# Inappropriate Off-label Use of a Qualitative, Point-of-care hCG Device

**DOI:** 10.5811/westjem.2016.10.32675

**Published:** 2016-12-06

**Authors:** Robert D. Nerenz, Ann M. Gronowski, David G. Grenache

**Affiliations:** * Dartmouth-Hitchcock Medical Center, Department of Pathology and Laboratory Medicine, Lebanon, New Hampshire; † Washington University School of Medicine, Department of Pathology and Immunology, St. Louis, Missouri; ‡ University of Utah School of Medicine, Department of Pathology, Salt Lake City, Utah

Dear Editor,

We read with interest the recent study by Gottlieb et al^1^ describing the reduction in turnaround time achieved by substituting whole blood for urine on a qualitative point-of-care (POC) hCG device. The device used in this study is FDA-approved and CLIA-waived only when the manufacturer’s instructions are followed: three drops of urine or serum are applied to the device and results are recorded within three minutes (urine) or five minutes (serum) after application of sample.^2^ However, the practice described by the authors differs considerably from the manufacturer’s instructions, as whole blood was used rather than urine or serum and results were interpreted after 10 minutes. Modification of an approved device constitutes off-label use, is considered a laboratory-developed test and requires extensive validation to establish the modified device’s performance characteristics before it is used in a clinical setting.

We commend the authors for noting that qualitative POC hCG devices are not FDA-approved for use with whole blood and we acknowledge their concurrent testing of urine on the same POC hCG device as a reference method. However, in addition to a method comparison study, CMS requires that laboratory-developed tests undergo an evaluation of precision, analytical sensitivity, analytical specificity, reportable range, reference interval and any other pertinent performance characteristics prior to being released for clinical use.^3^ Although a method comparison was performed, many additional device performance characteristics have not been defined. Furthermore, validation study results are limited to the specific clinical setting in which the study was performed and are not transferable to another institution, meaning that each institution that intends to offer a laboratory-developed test for clinical use must perform its own validation study. Use of an uncharacterized device to make clinical decisions puts patients at risk for adverse outcomes, particularly if inappropriate treatment is administered to a pregnant patient, an ectopic pregnancy goes undiagnosed due to a false negative result, or if necessary surgical intervention is delayed because of a false positive result. Use of modified devices without the required validation studies also jeopardizes the hospital laboratory’s accreditation and may result in forced discontinuation of laboratory testing, which negatively impacts patient care throughout the hospital.

We support the authors’ assertion that an FDA-approved device capable of rapid hCG detection in a whole blood specimen at the point of care would be valuable in healthcare delivery settings. We would like to point out that two FDA-approved test platforms are already available for exactly that: the Abbott i-STAT βhCG cartridge and the NowDiagnostics ADEXUSDx hCG test. In addition to receiving FDA approval, the performance characteristics of both of these devices have been independently evaluated in academic medical centers.^4^,^5^ We strongly recommend that the authors engage with laboratory professionals at their institution to discuss available testing options and select appropriate test methods that meet the clinical need without jeopardizing patient care.

## References

[1] Gottlieb M, Wnek K, Moskoff J (2016). Comparison of result times between urine and whole blood point-of-are pregnancy testing. West J Emerg Med.

[b2-wjem-18-324] 2Beckman Coulter Icon 25 hCG package intert, 2014.

[3] U.S. Government Publishing Office www.gpo.gov.

[4] Sowder AM, Yarbrough ML, Nerenz RD (2015). Analytical performance evaluation of the i-STAT total β-human chorionic gonadotropin immunoassay. Clin Chim Acta.

[5] Nerenz RD, Bell JR, de Oca NM (2016). Analytical and clinical evaluation of the NOWDiagnostics ADEXUSDx human chorionic gonadotropin test using whole blood. J App Lab Med.

