# Supplementary material for: Inappropriate Off-label Use of a Qualitative, Point-of-care hCG Device
Source: West J Emerg Med. 2016 Dec 6;18(2):324–5. doi: 10.5811/westjem.2016.10.32675 (PMC5321262; doi:10.5811/westjem.2016.10.32675)
Supplement: Related texts

# In reply to: “Inappropriate Off-label Use of a Qualitative, Point-of-care hCG Device”

**Published:** 2016-12-06

**Authors:** Michael Gottlieb, Kristopher Wnek, Jordan Moskoff, Errick Christian, John Bailitz

**Affiliations:** John H. Stroger Hospital of Cook County, Department of Emergency Medicine, Chicago, Illinois

Dear Dr. Robert D. Nerenz, Dr. Ann M. Gronowski, and Dr David G. Grenache,

Thank you for your comments regarding our recently published article describing a reduction in turnaround time achieved by the substitution of whole blood for urine on a qualitative point-of-care hCG device.^1^ We appreciate the insights and comments noted by Robert D. Nerenz, Ann M. Gronowski and David G. Grenache. The authors of this letter highlight the importance of multiple validation studies prior to routine implementation of non-FDA approved devices. We also agree with this and would like to highlight that the primary purpose of our study was to determine whether the substitution of whole blood for urine would decrease turnaround time, with the potential to reduce risks associated with delayed diagnoses of ectopic pregnancies, as well as expediting necessary imaging and treatment options that would be contingent upon pregnancy status.

While our study does support prior literature demonstrating similar accuracy between whole blood and urine for point-of-care hCG testing,^2^ our study clearly emphasizes that further study is necessary prior to routine acceptance. One of the primary goals of our article was to justify and encourage further study into this application in order to appropriately validate it for routine clinical use.

At the time of our study, there were no FDA-approved point-of-care hCG devices that could utilize whole blood. We were excited to hear of the FDA approval of two alternate point-of-care hCG devices for use with whole blood. While our study was the first to provide evidence of an advantage in turnaround times when using whole blood in place of urine, we look forward to further studies to determine whether similar results will be seen with these newer devices.

## References

[1] Gottlieb M, Wnek K, Moskoff J (2016). Comparison of result times between urine and whole blood point-of-are pregnancy testing. West J Emerg Med.

[2] Fromm C, Likourezos A, Haines L (2012). Substituting whole blood for urine in a bedside pregnancy test. J Emerg Med.
